# Emu oil offers protection in Crohn’s disease model in rats

**DOI:** 10.1186/s12906-016-1035-y

**Published:** 2016-02-06

**Authors:** Bhaskar Vemu, S. Selvasubramanian, V. Pandiyan

**Affiliations:** 1 Department of Veterinary Pharmacology and Toxicology, Madras Veterinary College, Chennai, Tamil Nadu 600 007 India; 2 Department of Veterinary Physiology and Biochemistry, Madras Veterinary College, Chennai, Tamil Nadu 600 007 India

**Keywords:** Aloe vera, Crohn’s disease, Emu oil, Indomethacin

## Abstract

**Background:**

Emu oil is a product of animal origin used for the treatment of inflammation, burns etc. as a part of aboriginal medicine in Australia. Crohn’s disease is a common inflammatory manifestation in humans and other animal species relating to the ulceration and digestive disturbances in upper gastro-intestinal tract. Aloe vera is commonly used substance from plant sources for inflammation, wound healing and various other properties. Given the difference in the source of the substances all the while playing a similar therapeutic role in different parts of the world, the present investigation was undertaken to evaluate the protective effect of aloe vera and emu oil alone and in combination; in comparison to sulfasalazine (Allopathic drug) as an alternative for the treatment of Crohn’s disease.

**Methods:**

Wistar albino rats were divided into six groups with two sub-groups of six animals each. After pre-treating the animals with sulfasalazine, aloe vera, emu oil and their combination for five consecutive days, the animals were sub-cutaneously administered indomethacin on 4^th^ and 5^th^ day and each sub-group was sacrificed on day 6 and 9. After sacrifice, serum and intestine of these animals was collected. Intestine length from duodenum till caecum was measured for estimating relative organ weight and disease activity index. Part of intestine was preserved in formalin for histopathology while the rest was used for analysis of oxidative parameters and myeloperoxidase. Serum collected was used for measuring alkaline phosphatase and cholesterol.

**Results:**

Assessment of the parameters in treatment groups indicated that the combination of aloe vera and emu oil resulted in better protection by suppressing the oxidative (P < 0.05) and histomorphological changes indicating a enhanced effect of these two agents which was found to be better than sulfasalazine.

**Conclusion:**

The combination of emu oil and aloe vera exhibited enhanced effect resulting in significant protection from indomethacin induced ulceration. This might be due to the different mechanism of anti-inflammatory effects (Salicylic acid in aloe vera and n3, n6 fatty acids acting as pseudosubstrates to cyclooxygenase enzyme) of components of the animal and plant products tested.

## Background

Crohn’s disease (CD) is a chronic condition observed in human and animal species affecting gastrointestinal tract. It is one of the forms of Inflammatory Bowel Disease (IBD) complex, while the other being Ulcerative Colitis (UC) [1]. Incidence of this condition, which doesn’t have a specific aetiology, has been on the increase owing to multitude of factors such as increased westernisation of habits (food and living), pollution, lowered immunity due to poor nutrition etc. [1]. Presently, the drugs used in the treatment of IBD are aimed at overall immunosuppression (corticosteroids) or mediator specific suppression (anti-TNF alpha, anti IL-6 antibodies etc.). Drugs used in the therapy of IBD include anti-inflammatory agents such as mesasalazine, sulfasalazine, corticosteroids, immunosuppressives (Azathioprine, 6-mercaptopurine), antibacterials (metronidazole, ornidazole, clarithromycin), biologics (infliximab, adalimumab, certolizumab pegol etc.) and probiotics (*Saccharomyces boulardii*, *Lactobacillus sp*, *Bifidobacterium sp*.) [2].

Other agents, such as plant and animal products, have been tested against IBD but with limited success. These include amla, wormwood, eicosapentaenoic acid, docosahexaenoic acid, fish oil, curcumin, linolenic acid etc. [3–6]. Rahimi et al. [7] reported some plants of Iranian origin having therapeutic effect against IBD, which include *Pistacia lentiscus*, *Commiphora mulkul*, *Terminalia chebula*, *Lepidum sativum* and *Althaea* sp. After perusal of the literature, it was found that aloe vera (*Aloe barbadensis* Miller) is one of the few plant products which has been known to offer moderate protection against IBD and has successfully entered clinical trials [8].

Emu oil was originally used by the aboriginals in Australia to treat pain and arthritic conditions. It is extracted from the retroperitoneal and sub-cutaneous fat of the flightless bird Emu (*Dromaius novaehollandiae*) [9]. Reports suggest that emu oil consists of high levels of unsaturated fatty acids (upto 50 %) such as oleic acid, linoleic acid, linolenic acid, palmitoleic acid etc. [10]. These fatty acids are also found in the liver oil of marine animals and were proven to be highly efficacious in treating inflammatory conditions [11]. Emu oil has been tested in arthritis, auricular, intestinal inflammation, hypercholesterolemia, burns etc. [9, 12–16].

Animal models for experimental replication of IBD can be spontaneous (cotton top tamarind model in monkeys), induced (Acetic acid/Oxazolone/Dextran Sulfate Sodium/TNBS) or through genetic manipulations (HLA-B_27_ transgenic rat/IL-2 deletion/IL-10 deletion/αβ TCR deletion/TGF-β_1_ deletion/Gi2α deletions in mice) [17]. Of these models due to the economic and ease of working and repeatability, chemically induced models are favoured. In induced models, TNBS and DSS are the commonly used models in mice whereas acetic acid and indomethacin models are commonly used in rats. Due to the ease of induction and the wide relevance of the model to clinical recurrence, indomethacin was used in the present study, which was reported to cause clinical condition that is similar in picture as well as mechanism to that of CD [18–20]. Several authors reported that the histomorphological picture of indomethacin induced inflammation has a similar picture to that of crohn’s disease indicating similar mechanisms. Also, indomethacin can induce as well as reactivate the clinical occurrence of IBD. Taking the above factors into consideration, the present study was planned to test the effect of aloe vera alone and in combination with emu oil against indomethacin induced CD [21].

## Methods

Indomethacin, emu oil and aloe vera gel extract were obtained from Tablets India Ltd., VR3 Emu farms & hatchery unit and Natural Remedies®, respectively. Sulfasalazine was procured from Sigma-Aldrich®, USA. All the chemicals used in the study were of analytical grade.

### Animals

Female wistar albino rats weighing 120-180 g were supplied by the Department of Laboratory animal medicine unit, Chennai. The animals were provided with ad libitum feed (M/S Tetragon Chemie Pvt. Ltd., Bangalore, India) and water. The experiments were carried out as per CPCSEA guidelines after obtaining the requisite approval from the Institutional Animal Ethics Committee (Lr.No1831/DFBS/E/IAEC/2010 dt.24.06.2011). Rats were divided into six groups with two sub groups of 6 animals each. First and second group served as healthy and negative control. The third group was administered sulfasalazine (100 mg/Kg body weight per os), fourth group – Aloe vera leaf gel extract (@ 400 mg/Kg body weight per os), fifth group – emu oil (@10 ml/Kg body weight per os) and sixth group – combination of aloe vera and emu oil (similar to the dose used in groups 4 & 5) for five consecutive days. Groups two through six were given sub-cutaneous indomethacin (5 %) prepared in 5 % sodium carbonate and administered at a dose rate of 10 mg.kg^-1^ on day 4 and 5 of study. On days six and nine, each sub group of animals across all groups were sacrificed. Intestine of these animals was collected from duodenum to caecum and washed with ice cold normal saline.

### Oxidative and inflammatory tests

The length and weight of the intestine along with body weight of the animal was measured for the estimation of DAI (Disease Activity Index) and ROW (Relative Organ Weight).

DAI = Weight of the intestine (g)/length of the intestine (cm)

ROW = Weight of the intestine (g)/body weight of the animal (g)

Macroscopic *s*coring was analysed by the method of Shirke [22]. Intestinal tissue was collected and stored at -20 °C till further analysis. The portion of intestine before the ileo-caecal junction was collected for homogenization (Heidolph Silent Crusher®, Germany) in phosphate buffer (pH 7.2, 50 mM). Homogenate (10 % W/V) was divided into three parts – one part mixed with 10 % trichloroacetic acid for measurement of reduced glutathione, other part for the measurement of lipid peroxidation and the third part was centrifuged at 15,000 g for 60 min at 4 °C and the supernatant was used for measurement of superoxide dismutase, catalase and glutathione peroxidase.

### Assay of lipid peroxidation (TBARS)

Thiobarbituric acid reactive substances were measured as per the procedure of Yagi [23]. Homogenate obtained was deproteinised using 10 % TCA followed by addition of 2 ml of thiobarbituric acid (46 mM thiobarbituric acid (TBA) in 1 N NaOH) and boiled for 30 min. Samples were centrifuged if found turbid. Absorbance was measured at an OD of 532 nm. TBARS was expressed as nM of MDA (Malonaldehyde) per gram of tissue.

### Assay of catalase

Catalase was measured as per the protocol of Caliborne [24]. Supernatant (0.2 ml) was added to 1 ml of hydrogen peroxide (30 mM hydrogen peroxide in 0.1 M phosphate buffer pH 7). Absorbance was measured for 3 min at a wavelength of 240 nm and the change in absorbance per minute was used to calculate activity using molar extinction co-efficient and activity is expressed as mM of hydrogen peroxide decomposed per minute per mg of protein.

### Assay of superoxide dismutase

Superoxide dismutase was measured according to Marklund and Marklund [25]. Assay mixture consisted of 2 ml of Tris-HCl (0.1 M, pH 8.2), 1.5 ml of distilled water and 0.5 ml of supernatant. Reaction was initiated by addition of 0.5 ml of pyrogallol (2 mM pyrogallol in 50 mM of Tris-HCl pH 7.4). A control has same assay mixture with addition of 2 ml distilled water instead of 1.5 ml in samples. Change in absorbance was recorded over 3 min period at a wavelength of 420 nm and change in absorbance per minute followed by enzyme activity which corresponds to amount of enzyme that inhibits auto-oxidation of pyrogallol by 50 % was calculated and expressed per mg of protein.

### Assay of glutathione peroxidase

It was assayed as per the method of Rotruck et al. [26]. Reaction mixture consists of 0.4 ml phosphate buffer (pH 7), 0.1 ml sodium azide (10 mM), 0.2 ml reduced glutathione (4 mM) and 0.2 ml of supernatant followed by 0.1 ml of 30 mM hydrogen peroxide finally making the total volume to 2 ml with distilled water. After 10 min incubation at room temperature, add 3 ml of disodium phosphate (0.3 M) and 1 ml of Dithiobis-2-nitrobenzoic acid (DTNB). Mix well and measure absorbance at a wavelength of 412 nm. Enzyme activity is expressed as micromoles of reduced glutathione utilised per min per milligram of protein.

### Assay of reduced glutathione

Reduced glutathione was measured as per Moron et al. [27]. It is based on the reaction of reduced glutathione with DTNB to give a compound that absorbs light at a wavelength of 412 nm. Method involves addition of 0.1 ml of homogenate with 0.9 ml of phosphate buffer (0.2 M, pH 8) followed by 2 ml (0.6 M DTNB) and measurement of the absorbance. It is expressed as μg of reduced glutathione per gram of tissue.

### Assay of myeloperoxidase

Estimation of myeloperoxidase was done as per the procedure of Krawisz et al. [28]. Half gram of sample was homogenised with 10 volumes of ice cold 50 mM Potassium phosphate buffer (pH 6) containing 0.5 % Hexadecyl Trimethyl Ammonium Bromide (HETAB). The resultant homogenate was subjected to sonication for 15 s, followed by centrifugation at 4^0^C, 12,000 g for 20 min. To the 0.1 ml supernatant, 2.9 ml of 50 mM phosphate buffer in 0.0005 % hydrogen peroxide (H_2_O_2_) was added. The change in absorbance over four minutes at a wavelength of 460 nm was observed from which the change in absorbance per minute was calculated. One unit of myeloperoxidase activity was defined as the change in absorbance per minute at room temperature.

Serum was separated for analysis of alkaline phosphatase (ALP) and cholesterol using Robonik® and Agappe® Diagnostics Ltd. kits as per manufacturer instructions. Intestine was stapled to cardboard to prevent shrinkage during tissue fixation in 10 % formalin. Sections were stained using Haematoxylin and Eosin stain [29]. All the values were expressed as Mean ± S.E. Values were statistically analysed using one way ANOVA followed by post-hoc analysis through tukey’s test using SPSS Version 20. *P* < 0.05 was considered significant.

## Results

### Composition of Emu oil

Emu oil was analysed using GC-MS and the fatty acid composition is presented in Table no. 1 [10].

### Macroscopic scoring, DAI, ROW, ALP and cholesterol

**Fig. 1:**
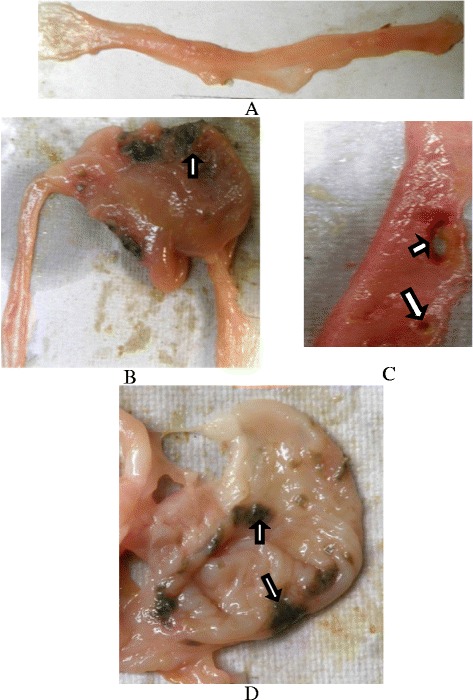
Macroscopic lesions of rat intestine (Caecum and Ileum). **a** Healthy control. **b** Necrosis of tissue in caecum of indomethacin treated rats (**c**) Longitudinal and pin point ulceration in ileum of indomethacin treated rats (**d**) Necrosis and granulation of caecum in sulfasalazine treated rats

The findings of the present investigation are tabulated in Tables 1, 2 and 3. Gross examination revealed no ulceration in control group but severe ulceration was noticed in indomethacin group which declined to some extent by day 9 of the study (Fig. 1). Interestingly, there were no ulcers in emu oil and combination groups on day nine. Upon macroscopic scoring it was evident that emu oil and combination groups had significant affect when compared to sulfasalazine group (Fig. 1 and Table 2). Similar pattern was observed in serum alkaline phosphatase levels along with DAI and ROW parameters, wherein there was significant elevation in indomethacin group throughout the study period but the treatment with emu oil and combination had significantly lowered it to near normal levels (Table 2). Since emu oil was being administered, serum cholesterol was analysed and no significant alterations developed during the study period.

**Table 1:** Composition of emu oil (GC-MS analysis)

Fatty acid	Percent (%)
Myristic acid (Saturated)	0.71
Palmitic acid (Saturated)	23.87
Stearic acid (Saturated)	10.76
Oleic acid (n9)	41.84
Linoleic acid (n6)	12.43
Linolenic acid (n3)	0.29
Palmitoleic acid (n7)	4.73
Total saturated fatty acid	35.34
Total unsaturated fatty acid	59.29

**Table 2:** Effect of the drug treatments on the relative organ weight, disease activity index, macroscopic scoring and serum biochemical parameters in indomethacin induced CD in rats

	Macroscopic scoring		ROW		DAI (g/cm)	
Group	day 6	day 9	day 6	day 9	day 6	day 9
Control	-	-	0.033^a^ ± 0.008	0.033^a^ ± 0.001	0.063^a^ ± 0.0013	0.061^a^ ± 0.0016
Indomethacin	2.20^d^ ± 0.20	1.43^b^ ± 0.20	0.054^b^ ± 0.002	0.056^d^ ± 0.001	0.092^b^ ± 0.003	0.082^b^ ± 0.0017
Sulfasalazine	2.00^cd^ ± 0.32	0.71^a^ ± 0.36	0.037^a^ ± 0.003	0.041^bc^ ± 0.001	0.0681^a^ ± 0.0013	0.063^a^ ± 0.0027
Aloe vera	1.60^bcd^ ± 0.25	0.50^a^ ± 0.22	0.048^ab^ ± 0.0006	0.049^c^ ± 0.0021	0.067^ab^ ± 0.0047	0.064^a^ ± 0.0016
Emu oil	1.00^abc^ ± 0.32	Nil	0.045^ab^ ± 0.004	0.039^ab^ ± 0.003	0.067^a^ ± 0.0033	0.064^a^ ± 0.0032
Aloe vera and emu oil combination	0.60^a^ ± 0.25	Nil	0.041^ab^ ± 0.0024	0.042^bc^ ± 0.001	0.077^ab^ ± 0.0062	0.063^a^ ± 0.0045
	Day 6			Day 9		
Group	Cholesterol (mg/dL)		ALP (U/L)	Cholesterol (mg/dL)	ALP (U/L)	
Control	63.24^a^ ± 2.14		103.23^a^ ± 1.01	68.45^a^ ± 6.58	106.77^a^ ± 12.37	
Indomethacin	61.74^a^ ± 3.045		272.28^c^ ± 25.18	49.80^a^ ± 5.42	274.33^c^ ± 11.74	
Sulfasalazine	50.69^a^ ± 9.23		182.67^b^ ± 23.76	52.36^a^ ± 1.53	224.40^bc^ ± 17.81	
Aloe vera	61.28^a^ ± 9.27		182.66^b^ ± 8.71	62.38^a^ ± 4.22	169.05^abc^ ± 41.01	
Emu oil	57.75^a^ ± 7.30		138.54^ab^ ± 8.12	64.35^a^ ± 4.65	153.50^ab^ ± 21.45	
Aloe vera and emu oil combination	56.44^a^ ± 9.48		177.32^b^ ± 20.09	54.98^a^ ± 8.44	154.60^ab^ ± 29.65	

All the values were expressed as Mean ± S.E. (n = 6) and means bearing different superscripts in a column differ significantly

**Table 3:** Effect of drug treatments on the intestinal tissue pro-oxidant and antioxidant parameters on day six of sacrifice in indomethacin induced CD in rats

Group	CAT	SOD	GP_X_	GSH	MDA	Myeloperoxidase
	(μM of H_2_O_2_ decomposed/min/mg protein)	(enzyme required to inhibit 50 % pyrogallol autoxidation/min/mg protein)	(μM of glutathione utilized/min/mg protein)	(mg/g of tissue)	(mM of MDA/g of tissue)	(U/mg of protein)
Control	8.73^b^ ± 0.01	6.15^b^ ± 0.55	5.17^c^ ± 0.49	2.85^a^ ± 0.25	54.58^d^ ± 1.16	15.40^a^ ± 0.84
Indomethacin	2.37^a^ ± 0.07	1.73^a^ ± 0.28	1.44^a^ ± 0.35	27.01^d^ ± 1.47	27.77^a^ ± 1.18	63.29^c^ ± 4.79
Sulfasalazine	6.12^ab^ ± 1.37	3.31^a^ ± 0.33	3.52^bc^ ± 0.22	11.90^c^ ± 0.59	31.83^ab^ ± 2.29	39.06^b^ ± 1.03
Aloe vera	4.26^ab^ ± 0.5	2.74^a^ ± 0.33	2.91^ab^ ± 0.19	10.96^c^ ± 0.58	34.75^ab^ ± 1.25	39.89^b^ ± 2.25
Emu oil	5.97^ab^ ± 0.50	2.95^a^ ± 0.44	3.67^bc^ ± 0.42	8.36^bc^ ± 0.91	41.71^bc^ ± 2.13	35.36^b^ ± 0.64
Aloe vera and emu oil combination	6.07^ab^ ± 1.02	3.22^a^ ± 0.26	3.97^bc^ ± 0.68	5.92^ab^ ± 1.40	46.58^cd^ ± 3.06	33.66^b^ ± 4.14

All the values were expressed as Mean ± S.E. (n = 6) and means bearing different superscripts in a column differ significantly

### Oxidative status, myeloperoxidase and histopathology

**Fig. 2:**
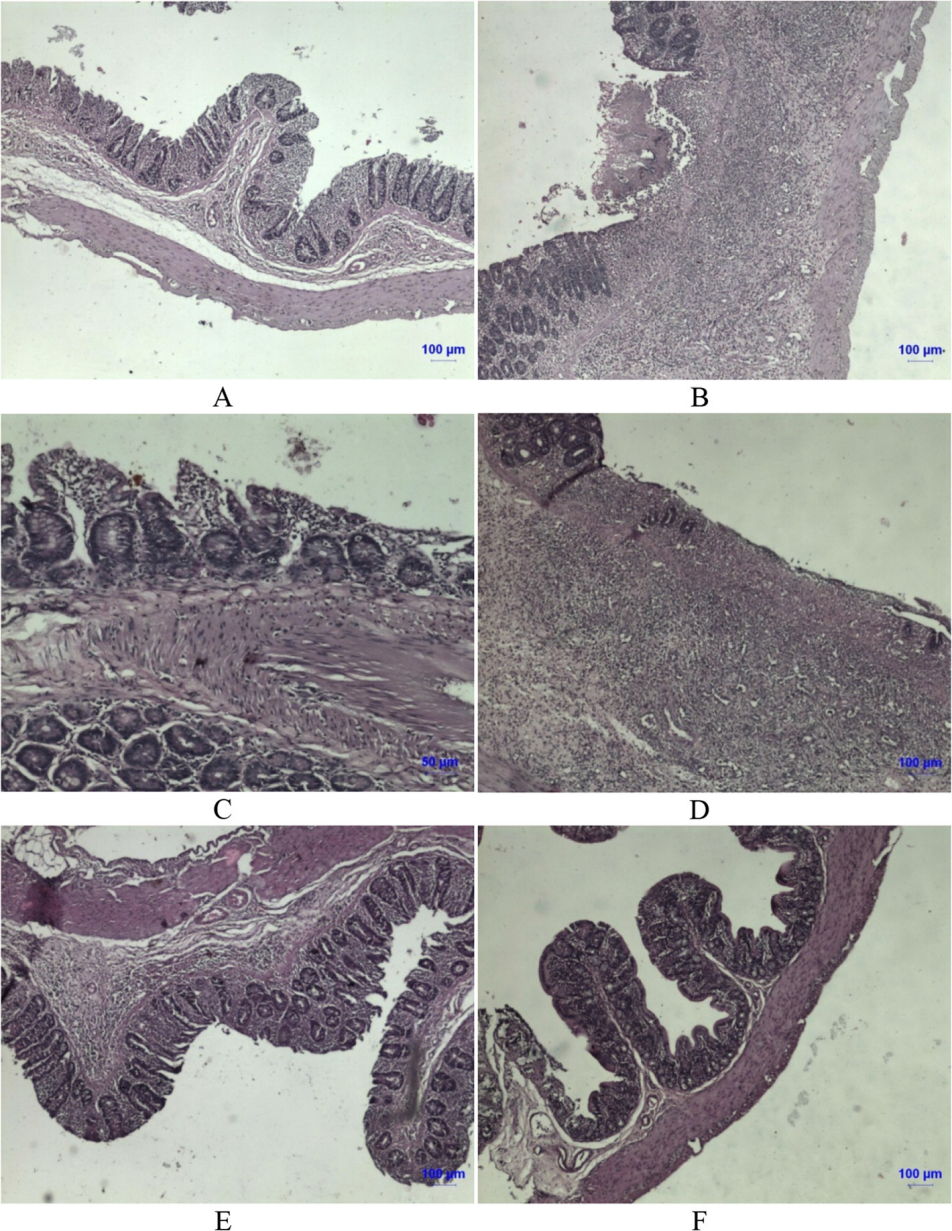
Microscopic picture in sections obtained from ileo-caecal region in rats sacrificed on day nine. **a** Healthy control (**b**) Ulceration, loss of villi, infiltration of neutrophils and eosinophilic staining of lamina propria in indomethacin treated rats (**c**) Loss of villi architecture and infiltration of neutrophils in sulfasalazine treated rats (**d**) Loss of caecal glands, ulceration and eosinophilic staining of lamina propria in aloe vera treated rats (**e**) Presence of caecal glands and partial loss of epithelium observed in emu oil treated rats (**f**) Normal caecal architecture with villi projections and normal staining of lamina propria in rats treated with the combination of aloe vera and emu oil

Assessment of the oxidative parameters indicated an improvement in the overall antioxidant state of intestine in emu oil and combination groups as compared to aloe vera and sulfasalazine groups, which in turn were better than the indomethacin group (Tables 3 and 4). Intestines of control group were normal with presence of goblet cells and villi architecture (Fig. 2). Noticeable changes in the neutrophil infiltration, transudate accumulation in lamina propria, denudation of epithelium etc. have been observed in indomethacin group. These changes have been prevented to some extent by treating with sulfasalazine and aloe vera as observed in histopathological sections but neutrophil infiltration was still severe as indicated by dense cellularity of lamina propria and eosinophilic staining indicative of inflammatory exudate. On the other hand, in the groups treated with emu oil and combination, the degenerative changes were least along with trace presence of goblet cells and villi indicating superior protection from the damage caused by indomethacin. Emu oil and its combination with aloe vera appeared to have better effect among all the treatment groups.

**Table 4:** Effect of the drug treatments on the intestinal tissue pro-oxidant and antioxidant parameters on day nine of sacrifice in indomethacin induced CD in rats

Group	CAT	SOD	GP_X_	GSH	MDA	Myeloperoxidase
	(μM of H_2_O_2_ decomposed/min/mg protein)	(enzyme required to inhibit 50 % pyrogallol autoxidation/min/mg protein)	(μM of glutathione utilized/min/mg protein)	(mg/g of tissue)	(mM of MDA/g of tissue)	(U/mg of protein)
Control	8.53^c^ ± 0.29	6.46^b^ ± 0.55	5.70^c^ ± 0.4	2.39^a^ ± 0.29	56.68^c^ ± 2.48	16.63^a^ ± 1.85
Indomethacin	3.11^a^ ± 0.31	1.67^a^ ± 0.166	1.56^a^ ± 0.08	30.62^c^ ± 4.28	24.62^a^ ± 1.72	46.65^c^ ± 2.27
Sulfasalazine	6.11^abc^ ± 0.94	3.47^ab^ ± 0.19	3.25^ab^ ± 0.52	13.68^b^ ± 1.92	31.49^ab^ ± 3.02	25.46^ab^ ± 1.77
Aloe vera	4.35^ab^ ± 0.52	3.20^a^ ± 0.18	2.47^a^ ± 0.19	15.82^b^ ± 0.99	36.76^ab^ ± 1.59	32.65^b^ ± 2.64
Emu oil	6.26^abc^ ± 0.74	3.75^ab^ ± 0.16	4.48^bc^ ± 0.69	8.93^ab^ ± 0.63	44.75^bc^ ± 2.45	30.85^b^ ± 3.80
Aloe vera and emu oil combination	6.91^bc^ ± 0.18	4.55^ab^ ± 0.15	4.95^bc^ ± 0.12	8.33^ab^ ± 0.80	37.66^bc^ ± 5.23	29.68^b^ ± 2.07

All the values were expressed as Mean ± S.E. (n = 6) and means bearing different superscripts in a column differ significantly

## Discussion

Rats were treated with indomethacin to induce CD like condition and the efficacy of aloe vera and emu oil in comparison to sulfasalazine was evaluated. Indomethacin administration resulted in ulceration of intestine in caecum and ileum. The process of ulceration can be divided into topical and resolution phases. In the former, sloughing of mucus and epithelial layers along with reduction in the protection offered by phosphatidyl choline is observed [30–33]. NSAIDs (Non steroidal anti-inflammatory drugs) could remove or replace phosphatidyl choline leading to production of simple toxic or super toxic micelles. Due to entrapment and accumulation of indomethacin in epithelial cells, uncoupling of oxidative phosphorylation, reduction of ATP levels, altered Ca^++^ homeostasis, ROS (Reactive Oxygen Species) production and cell death results [34]. It has been reported to alter blood circulation leading to venous stasis, stunting of villi and accumulation of neutrophils, ultimately resulting in ulceration and absorption of endotoxins released by enteric bacteria, further intensifying the inflammation [35].

In the resolution phase, due to inhibition of COX-1 enzymes there is reduction in the synthesis of anti-inflammatory mediators. It has been reported that COX-1 expression is highest in small intestine of humans and any non-selective NSAID mediated injury was was restricted to upper gastrointestinal tract in humans indicating the role of COX-1 in resolution phase, required for synthesis of anti-inflammatory prostaglandins. The NSAID mediated injury in rats was limited to lower GI tract, due to differences in the distribution of COX-1 enzyme [36].

DAI and ROW parameters are based on body weight, intestine weight and length. CD might hamper nutrient absorption ensuing in weight reduction. Since, the present study is for a short duration, ratio of organ to body weight has been considered assuming that the body weight has been normalised between different groups. Any reduction in body weight and increase in weight of intestine (accumulation of fluids due to inflammation) would increase ROW. Similarly, inflammation would result in shortening of intestine and increase in DAI. ALP is a non specific marker of intestine and any increase in control conditions are indicative of intestinal affection. Cholesterol, a major indicator of lipid absorption did not show any significant changes even after administration of emu oil, which might be due to the reduction in the bile flow [37].

Myeloperoxidase is an enzyme present in the neutrophils, which helps in the production of hypochlorous acid (HOCl) that has antibacterial properties and depolymerises gastrointestinal mucin, peroxidise lipid and protein [38] through the release of hydroxyl and chloride radicals resulting in oxidative stress [39]. It also has chemotactic activity, thereby increasing the inflammatory cell population and intensifying oxidative stress. Aloe vera and emu oil, through their antioxidant activity might have resulted in combating these free radicals thereby offering protection to intestine. Any amount of oxidative stress would be perilous to intestine as it is one of those organs which have poor defensive mechanisms against oxidative stress [40].

It is established that sulfasalazine acts on NF-kB (Nuclear Factor Kappa B) gene and has anti-inflammatory and antioxidant activity [41]. Similarly, fractions of emu oil might act at gene level and down regulate NF-kB resulting in anti-inflammatory activity as reported by Borniquel and co-workers for nitrated oleic acid in IBD [42]. Once inflammatory cell migration and tissue injury are inhibited overall antioxidant status of intestine gets improved. Even though there was reduction in the number of ulcers in emu oil and combination groups on day nine, there were multiple microscopic ulcerations in sections of all groups except group I.

The mechanism of anti-inflammatory activity of emu oil and aloe vera might be through intervening at multiple steps in the process of initiation and intensification of intestinal inflammation. Aloe vera has various components that act as antioxidant and anti-inflammatory agents. These include alprogen that inhibits mast cell degranulation thereby inhibiting the release of histamine and leukotrienes [43], along with salicylic acid and β-sitosterol which have anti-inflammatory activity [44]. Similarly, PUFA’s in emu oil could act as false substrates to inflammatory enzymes resulting in synthesis of prostaglandins, leukotrienes and resolvins. Prostaglandins (3 series) and leukotrienes (5 series) have poor pro-inflammatory activity whereas resolvins have been reported to possess anti-inflammatory activity through ChemR23 (G protein coupled receptor involved in inflammation) [45]. Anti-inflammatory effect of emu oil could also be attributed to its role in inhibiting indomethacin accumulation, translocation of intestinal biota to lamina propria, providing antioxidant activity etc. Emu oil composition too indicates that higher levels of unsaturated fatty acids are present leading to faulty synthesis of the pro-inflammatory mediators with weak pro-inflammatory and potent anti-inflammatory activities. So, aloe vera and emu oil when administered simultaneously would act at different steps of inflammation resulting in wholesome effect.

## Conclusion

In the present scenario, finding a specific molecule for the treatment of idiopathic conditions is difficult. A wholesome agent that can act at various levels of the inflammatory process is required. This is possible to some extent vide the use of complex agents such as plant extracts or oils (animal and/or vegetable origin) in encapsulated form for drug delivery. The present investigation has revealed an interesting fact, that through simultaneous use of emu oil and aloe vera gel extract, a enhanced effect in the therapy of indomethacin induced CD model is produced, which is perceptible through biochemical and oxidative parameters. This study therefore corroborates the use of emu oil and aloe vera for intestinal inflammation as an alternative medicine and suggests the use of encapsulated preparations for better therapeutic effect.

## Abbreviations

ALP: Alkaline phosphatase
ANOVA: Analysis of variance
ATP: Adenosine triphosphate
CD: Crohn’s disease
COX: Cyclo-oxygenase
CPCSEA: Committee for the purpose of control and supervision for experimentation on animals
DAI: Disease activity index
GI: Gastro-intestinal
IBD: Inflammatory Bowel Disease
NF-kB: Nuclear Factor Kappa B
NSAID: Non steroidal anti-inflammatory drugs
ROS: Reactive Oxygen Species
ROW: Relative Organ Weight
S.E.: Standard error
UC: Ulcerative colitis

## References

[1] Fiocchi C (1998). Inflammatory Bowel Disease: etiology and pathogenesis. Gastroenterology.

[2] Triantafillidis KJ, Merikas E, Georgopoulos F (2011). Current and emerging drugs for the treatment of inflammatory bowel disease. Drug Des Dev Ther.

[3] Deshmukh CD, Pawar AT, Bantal V (2010). Effect of *Embilica officinalis* methanolic fruit extract on indomethacin induced enterocolitis in rats. Res J Med Plant.

[4] Krebs S, Omer TN, Omer B (2010). Wormwood (*Artemisia absinthium*) suppresses tumor necrosis factor alpha and accelerates healing in patients with Crohn’s disease – A controlled clinical trial. Phytomedicine.

[5] Jia Q, Ivanov I, Zlatev ZZ, Alaniz RC, Weeks BR, Callaway ES (2011). Dietary fish oil and curcumin combine to modulate colonic cytokinetics and gene expression in dextran sodium sulphate treated mice. Br J Nutr.

[6] Neilson AP, Djuric Z, Ren J, Hong YH, Sen A, Lager C (2012). Effect of cyclooxygenase genotype and dietary fish oil on colonic eicosanoids in mice. J Nutr Biochem.

[7] Rahimi R, Ardekani MRS, Abdollahi M (2010). A review of the efficacy of traditional Iranian medicine for inflammatory bowel disease. World J Gastroenterol.

[8] Park MY, Kwon HJ, Sung MK (2011). Dietary aloin, aloesin, or aloe-gel exerts anti-inflammatory activity in a rat colitis model. Life Sci.

[9] Whitehouse MW, Turner AG, Davis CKC, Roberts MS (1998). Emu Oil(s): a source of non-toxic transdermal anti-inflammatory agents in aboriginal medicine. Inflammopharmacology.

[10] Vemu B, Selvasubramanian S, Pandiyan V. Anti-inflammatory activity of emu oil in indomethacin induced inflammatory bowel disease in rats. Proc Indian Natl Sci Acad B Biol Sci. 2015; doi: 10.1007/s40011-015-0564-3.

[11] Khare S, Asad M, Dhamanigi SS, Satya PV (2008). Antiulcer activity of cod liver oil in rats. Ind J Pharmacol.

[12] Yoganathan S, Nicolosi R, Wilson T (2003). Antagonism of croton oil inflammation by topical emu oil in CD-1 mice. Lipids.

[13] Lagniel C, Torres AM (2007). Consequences of burn injuries treatment with 100 % pure emu oil. Burns.

[14] Wilson AT, Nicolosi RJ, Handelman G, Yoganathan S, Kotyla T, Orthoefer F (2004). Comparative effects of emu and olive oil on aortic early atherosclerosis and associated risk factors in hypercholesterolemic hamsters. Nutr Res.

[15] Abimosleh SM, Lindsay RJ, Butler RN, Cummins AG, Howarth GS (2011). Emu oil increases colonic crypt depth in a rat model of ulcerative colitis. Dig Dis Sci.

[16] Abimosleh SM, Tran CD, Howarth GS (2012). Emu Oil: a novel therapeutic for disorders of the gastrointestinal tract?. J Gastroenterol Hepatol.

[17] Sartor RB (1995). Insights into the pathogenesis of inflammatory bowel diseases provided by New Rodent Models of spontaneous colitis. Inflamm Bowel Dis.

[18] Yamada T, Deitch E, Specian RD, Perry MA, Sartor RB, Grisham MB (1993). Mechanisms of acute and chronic intestinal inflammation induced by indomethacin. Inflammation.

[19] Anthony A, Pounder RE, Dhillon AP, Wakefield AJ (2000). the inflammatory bowel disease study group. Similarities between ileal Crohn’s disease and indomethacin experimental jejunal ulcers in the rat. Aliment Pharmacol Therapeut.

[20] Bonner GF, Fakhri A, Vennamaneni SR (2004). A long term cohort study of non steroidal anti-inflammatory drug use and disease activity in outpatients with inflammatory bowel disease. Inflamm Bowel Dis.

[21] Langmead L, Feakins RM, Goldthorpe S, Holt H, Tsironi E, De Silva A, et al. Randomized, double-blind, placebo-controlled trial of oral aloe vera gel for active ulcerative colitis. Aliment Pharmacol Therapeut. 2004b;19:739-47. 10.1111/j.1365-2036.2004.01902.x 15043514

[22] Shirke SS (2011). Effect of polyherbal formulation on experimental models of inflammatory bowel diseases, M. Pharm. Thesis, Mumbai University, Mumbai, 2002. In: Jagtap AG, Niphadkar PV. Protective effect of *Bombax malabaricum* DC on experimental models of inflammatory bowel disease in rats and mice. Indian J Exp Biol.

[23] Yagi K (1976). Simple fluorimetric assay for lipid peroxides in blood plasma. Biochem Med.

[24] Caliborne AL, Greenwald RA (1985). Assay of catalase. Handbook of Oxygen Radical Research.

[25] Marklund SL, Marklund G (1974). Involvement of superoxide anion radical in the autooxidation of pyrogallol and a convenient assay for superoxide dismutase. Eur J Biochem.

[26] Rotruck JD, Pope AL, Ganther HE, Swanson AB, Hafeman DG, Hoekstra WG (1973). Selenium, biochemical role as a component of glutathione peroxidase and assay. Science.

[27] Meron MS, Depierre JW, Mannervik B (1979). Levels of glutathione, glutathione reductase and glutathione-s-transferase activities in rat lung and liver. Biochim Biophys Acta.

[28] Krawisz JE, Sharon P, Stenson WF (1986). Quantitative assay for acute intestinal inflammation based on myeloperoxidase activity. Assessment of inflammation in rat and hamster models. Gastroenterology.

[29] Ashtaral NL, Mohammadirad A, Yasa N, Minaie B, Nikfar S, Ghazanfari G (2006). Benefits of *Zataria multiflora* Boiss in experimental model of mouse inflammatory bowel disease. eCAM.

[30] Kelfalakes H, Stylianides TJ, Amanakis G, Kolios G (2009). Exacerbation of inflammatory bowel diseases associated with the use of non-steroidal anti-inflammatory drugs: myth or reality?. Eur J Clin Pharmacol.

[31] Barrios JM, Litchinberger LM (2000). Role of Biliary phosphatidyl choline in bile acid protection and NSAID injury of the ileal mucosa in rats. Gastroenterology.

[32] Lichtenberger LM, Wang ZM, Romero JJ, Ulloa C, Perez JC, Giraud MN (1995). Non-steroidal anti-inflammatory drugs (NSAIDs) associate with zwitterionic phospholipids: Insight into the mechanism and reversal of NSAID induced gastrointestinal injury. Nat Med.

[33] Somasundaram S, Rafi S, Hayllar J, Sigthorsson G, Jacob M, Price AB (1997). Mitochondrial damage: a possible mechanism of the “topical” phase of NSAID induced injury to the rat intestine. Gut.

[34] Petruzzelli M, Vacca M, Moschetta A, Cinzia SR, Palasciano G, van Erpecum KJ (2007). Intestinal mucosal damage caused by non-steroidal anti-inflammatory drugs: Role of bile salts. Clin Biochem.

[35] Kelly D, Anthony A, Piasecki C, Lewin J, Pounder RE, Wakefield AJ (2000). Endothelial changes precede mucosal ulceration induced by indomethacin: an experimental study in the rat. Aliment Pharmacol Therapeut.

[36] Radi ZA, Khan NK (2006). Effects of cyclooxygenase inhibition on the gastrointestinal tract. Exp Toxicol Pathol.

[37] Yamada T, Hoshino M, Hayakawa T, Kamiya Y, Ohhara H, Mizuno K (1996). Bile secretion in rats with indomethacin-induced intestinal inflammation. Am J Physiol.

[38] Conner EM, Brand SJ, Davis JM, Kang DY, Grisham MB (1996). Role of reactive metabolites of oxygen and nitrogen in inflammatory bowel disease: toxins, mediators, and modulators of gene expression. Inflamm Bowel Dis.

[39] Klebanoff SJ, Gallin JI, Goldstein IM, Snyderman R (1992). Oxygen metabolites from phagocytes. Inflammation: basic principles and clinical correlates.

[40] Buffinton GD, Doe WF. Altered ascorbic acid status in the mucosa from inflammatory bowel disease patients. Free Radic Biol Med. 1995a;22:131-43. 10.3109/10715769509147535 7704184

[41] Wahl C, Liptay S, Adler G, Schmid RM (1998). Sulfasalazine: a potent and specific inhibitor of nuclear factor kappa B. J Clin Investig.

[42] Borniquel S, Jansson EA, Cole MP, Freeman BA, Lundberg JO (2010). Nitrated oleic acid up-regulates PPARγ and attenuates experimental inflammatory bowel disease. Free Radic Biol Med.

[43] Ro JY, Lee B, Kim JY, Chung Y, Chung MH, Lee SK (2000). Inhibitory mechanism of aloe single component (Alprogen) on mediator release in guinea pig lung mast cells activated with specific antigen-antibody reactions. J Pharmacol Exp Therapeut.

[44] Surjushe A, Vasani R, Saple DG (2008). Aloe Vera: a short review. Indian J Dermatol.

[45] Arita M, Bianchini F, Aliberti J, Sher A, Chiang N, Hong S (2005). Stereochemical assignment, anti-inflammatory properties and receptor for the omega-3 lipid mediator resolvin E1. J Exp Med.

